# Different Extracellular β-Amyloid (1-42) Aggregates Differentially Impair Neural Cell Adhesion and Neurite Outgrowth through Differential Induction of Scaffold Palladin

**DOI:** 10.3390/biom12121808

**Published:** 2022-12-02

**Authors:** Tianyu Zhang, Chuli Song, He Li, Yanru Zheng, Yingjiu Zhang

**Affiliations:** 1 Key Laboratory for Molecular Enzymology and Engineering of the Ministry of Education, Jilin University, Changchun 130012, China; 2 School of Life Science, Jilin University, Changchun 130012, China

**Keywords:** Alzheimer disease, Aβ42, adhesion, neurite outgrowth, palladin, extracellular matrix

## Abstract

Extracellular amyloid β-protein (1-42) (Aβ42) aggregates have been recognized as toxic agents for neural cells in vivo and in vitro. The aim of this study was to investigate the cytotoxic effects of extracellular Aβ42 aggregates in soluble (or suspended, SAβ42) and deposited (or attached, DAβ42) forms on cell adhesion/re-adhesion, neurite outgrowth, and intracellular scaffold palladin using the neural cell lines SH-SY5Y and HT22, and to elucidate the potential relevance of these effects. The effect of extracellular Aβ42 on neural cell adhesion was directly associated with their neurotrophic or neurotoxic activity, with SAβ42 aggregates reducing cell adhesion and associated live cell de-adherence more than DAβ42 aggregates, while causing higher mortality. The reduction in cell adhesion due to extracellular Aβ42 aggregates was accompanied by the impairment of neurite outgrowth, both in length and number, and similarly, SAβ42 aggregates impaired the extension of neurites more severely than DAβ42 aggregates. Further, the disparate changes of intracellular palladin induced by SAβ42 and DAβ42 aggregates, respectively, might underlie their aforementioned effects on target cells. Further, the use of anti-oligomeric Aβ42 scFv antibodies revealed that extracellular Aβ42 aggregates, especially large DAβ42 aggregates, had some independent detrimental effects, including physical barrier effects on neural cell adhesion and neuritogenesis in addition to their neurotoxicity, which might be caused by the rigid C-terminal clusters formed between adjacent Aβ42 chains in Aβ42 aggregates. Our findings, concerning how scaffold palladin responds to extracellular Aβ42 aggregates, and is closely connected with declines in cell adhesion and neurite outgrowth, provide new insights into the cytotoxicity of extracellular Aβ42 aggregates in Alzheimer disease.

## 1. Introduction

Alzheimer disease (AD) is a progressive neurodegenerative disease, and amyloid β-protein with 42 amino acids (Aβ42) is a key molecule involved in AD pathogenesis. Typical histopathological features of AD are extracellular Aβ42 aggregates and dense amyloid plaques in the human brain, especially in the hippocampus [1,2]. Aβ42 aggregates, especially Aβ42 oligomers, have been recognized as extracellular toxic agents for neural cells, and they directly disrupt the normal interaction between neural cells and the extracellular matrix (ECM) or other cells in vivo and in vitro [3]. Further, these metastable and heterogeneous Aβ42 aggregates, whether soluble (or suspended) or deposited, will gradually disrupt the normal metabolic pathways of neural cells, eventually leading to neural cell damage and loss [4,5]. Therefore, Aβ42 aggregates are central to AD pathogenesis.

The normal growth and function of cells, especially neural cells, require the support, buffering, and nourishment of a specific extracellular environment [6,7]. In addition to this, ECM plays a vital role in neuron survival and growth, including neural cell adhesion and neurite outgrowth (neuritogenesis) [8,9]. Neural cell adhesion is an attachment behavior to the extracellular matrix or other cells; it is essential for neurite outgrowth, a prerequisite for the formation of synapses between neurons and their former targets and the communication between neural cells [8,9,10,11]. Neural cell adhesion and neurite outgrowth play well-established roles in building and maintaining synaptic structure, including synapse morphogenesis and plasticity (synaptogenesis), and eventually affects neurogenesis [12,13]. Neural cell adhesion and neurite outgrowth are affected not only by their extracellular environment, but also by their intracellular regulation, allowing for adjustments to a changing extracellular environment [14].

Our recent report shows that different species (monomers, oligomers, and fibrils) and forms (soluble/suspended or deposited/attached) of extracellular Aβ42 influence the migration of target cells to different degrees, and that Aβ42 aggregates in ECM serve as adverse “anchors” that make neural cells inert. Migration, adhesion, and neurite outgrowth are three closely related functional behaviors of neural cells, all of which are primarily based on organizing and/or remodeling of the actin cytoskeleton besides microtubules [15,16]. The organization and/or remodeling of the actin cytoskeleton is regulated by a variety of scaffold proteins, among which the scaffold protein palladin is essential for neural cells [17,18]. Palladin exists widely in neural cells and is required to maintain the integrity of the actin cytoskeleton and, thus, the neurite morphology of neural cells. Therefore, in a variety of neural cells, palladin functions as an actin binding and bundling protein and plays an important role in normal adhesion, neuritogenesis, and neuronal maturation [19]. More importantly, palladin is directly involved in regulating the stress response to ECM, which is essential for neurite occurrence and extension [20]. However, whether and how palladin affects neurite outgrowth in the presence of extracellular Aβ42 remains unknown, although previous studies have shown that palladin plays a key role in neurite outgrowth in cultured neurons [19,21].

Both adhesion and neurite outgrowth involves various interactions between cells and ECM, which are accordantly affected by the properties and/or components of the extracellular environment [22]. However, in many neurological diseases, especially neurodegenerative diseases such as AD, little is known about how the adhesion of neural cells and neurite outgrowth are affected by various Aβ42 species (soluble/suspended or deposited Aβ42 oligomers and high aggregates) in ECM, and whether extracellular Aβ42 can stimulate the response of intracellular scaffold proteins associated with adhesion and neurite outgrowth. Therefore, investigating the effects of these extracellular Aβ42 species can provide information on adhesion- and neuritogenesis-related cellular events, and on the heterogeneity and complexity of the underlying mechanisms of these extracellular Aβ42 species, to gain a more comprehensive understanding of the toxic activity of Aβ42 aggregates in ECM. In this study, we aimed to investigate the effects of these extracellular Aβ42 species on neural cell adhesion, neurite outgrowth, and cellular scaffold palladin, and to elucidate the potential relevance of these effects.

## 2. Materials and Methods

### 2.1. Aβ42 and Its Aggregates

Human Aβ42 protein was purchased from Dalian Meilun Biological Co., Ltd. (MB10425, Dalian, China). Aβ42 monomer (Aβ42M) solution was prepared as described previously [23]. Aβ42 oligomers (Aβ42O) and fibrils (Aβ42F) were prepared from Aβ42M as described previously [24]. The three Aβ42 species in soluble (or suspended) and deposited (or attached) forms (S/DAβ42M, S/DAβ42O, and S/DAβ42F) were used in this study to analyze their effects on neural cell adhesion, neurites, and the intracellular scaffold palladin.

### 2.2. Cell Culture

Human neuroblastoma cell line SH-SY5Y (338056, BeNa Culture Collection, Beijing, China) and mouse primary hippocampal neuronal cell line HT22 (337709; BeNa Culture Collection, Beijing, China) were used in this study. Unless otherwise stated, the cell lines were cultured in Dulbecco’’s Modified Eagle Medium (DMEM) (11965092, GIBCO, Shanghai, China) containing 10% FBS (16140071, GIBCO, Shanghai, China), 100 U/mL penicillin, and 100 µg/mL streptomycin (15140148, GIBCO, Shanghai, China) in a humidified atmosphere of 5% CO_2_ at 37 °C for a specified period of time. All the cells used in this study were between passages 4 and 6.

Differentiated SH-SY5Y or HT22 cells were prepared by gradually removing heat-inactivated fetal bovine serum (hiFBS) and Eagle’s minimum essential medium (EMEM) (MEP12-10LT, Caisson, Shanghai, China) and sequentially introducing glutamine (G0200, Solarbio, Beijing, China), all-trans retinoic acid (RA) (207340010, GIBCO, Shanghai, China), brain-derived neurotrophic factor (BDNF) (P00062, Solarbio, Beijing, China), neurobasal medium (21103049, GIBCO, Shanghai, China), B-27 supplement (17504001, GIBCO, Shanghai, China), and dibutyryl cyclic AMP (H31022649, First Biochemical Pharmaceutical Co., Ltd., Shanghai, China), with little modification to what has previously been described [25]. Briefly, after undifferentiated cells were generally cultured for 24 h and reached approximately between 50 and 70% confluency, the cells were rinsed with 1× phosphate-buffered saline (PBS) and were cultured sequentially with basic growth medium, differentiation media #1, #2, and #3 (see recipe below), at 37 °C with 5% CO_2_, which allowed SH-SY5Y cells to differentiate to a neuronal phenotype with more expansive and branched neurites. When the differentiated cells reached approximately 70% confluency, they could be used for subsequent biochemical and imaging analyses.

Basic growth medium: EMEM with 15% hiFBS, 0.01×Pen/Strep (1719675, GIBCO, Shanghai, China), 20 μM Glutamine.

Differentiation medium #1: 2.5% hiFBS, 0.01 × Pen/Strep, 20 μM Glutamine, and 20 nM RA.

Differentiation medium #2: EMEM with 1% hiFBS, 0.01 × Pen/Strep, 20 μM Glutamine, 20 nM RA.

Differentiation media #3: Neurobasal medium with 0.02 × B-27, 0.4 mM KCL, 0.01 × Pen/Strep, 20 μM Glutamine, 0.25 ng/mL BDNF, 4 μM dibutyryl cyclic AMP, and 20 nM RA.

### 2.3. Determination of Cell Adhesion and Re-Adhesion in the Presence of Various Extracellular Aβ42 Species

The adhesion and re-adhesion rates were determined using SH-SY5Y or HT22 cells by MTT assay. In the group using Aβ42-containing DMEM medium, SH-SY5Y or HT22 cells were seeded in 96-well plates at a density of approximately 8 × 10^4^ cells per well and cultured with 10% DMEM medium at 37 °C with 5% CO_2_ from 4–6 h to enable attachment to the bottom of wells. Subsequently, the culture medium was aspirated and fresh 10% DMEM medium containing SAβ42 or its aggregates (final concentration: 0.02–2.0 μM) with or without anti-oligomeric Aβ42 single-chain variable fragment (scFv) HT7 (or HT6) antibody (final concentration: 2.0 μM) [26,27] was added to each well. Cells were cultured at 37 °C with 5% CO_2_ for 12 h. The culture medium was collected, and the adhering cells were washed twice using 10 mM PBS (pH 7.2–7.4) and the washes were collected. After centrifugation (22 °C, 210× *g*, 5 min), the shed (debonded) cells in the medium and washes were collected together and were re-seeded into another 96-well plate. The re-seeded cells were incubated with fresh 10% DMEM medium at 37 °C with 5% CO_2_ from 4–6 h to allow the living cells to re-attach to the bottom of the well. Finally, the total numbers of the adherent cells and re-adherent cells (the cells that de-adhered alive and were capable of re-adhering or re-attaching to the bottom) were determined using conventional MTT assay, as described below. Each experiment was performed in at least triplicate and repeated in nine different batches of cells.

In the group using Aβ42-coated (or attached) plates, Aβ42 or its aggregates (final concentration: 0.02–2.0 μM) were initially added to 96-well plates and incubated at 4 °C overnight for deposition (or attachment) to the bottom of wells. Subsequently, the supernatant was aspirated and cells were seeded into the Aβ42-deposited plate at an approximate density of 8 × 10^4^ cells per well, followed by the addition of the fresh 10% DMEM medium with or without HT7 (or HT6) antibody (final concentration: 2.0 μM) to each well. Cells were cultured at 37 °C with 5% CO_2_ for 12 h. Similarly, the adhering cells were washed twice using 10 mM PBS (pH 7.2–7.4) and the shed cells in the medium and washes were collected and cultured with fresh 10% DMEM medium at 37 °C with 5% CO_2_ from 4–6 h as described above. Finally, the total numbers of the adhered and re-adhered cells were determined using conventional MTT assay, as described below. Each experiment was performed in at least triplicate and repeated in nine different batches of cells.

MTT assay was performed by following conventional methods. Briefly, the spent culture medium was aspirated and fresh 1% DMEM medium and 20 μL of MTT (M1020, Solarbio, Beijing, China) were sequentially added into each well, followed by incubation at 37 °C with 5% CO_2_ for another 4 h. The supernatant was discarded and 150 μL of DMSO was added into the culture for dissolving MTT completely. After 30 sec of oscillation, the absorbance of the culture sample was measured at 490 nm using a Thermo Microplate reader (TECAN, Shanghai, China).

The cells in the complete control group (Ctrl0) were cultured at 37 °C, 5% CO_2_ from 12–16 h, and the total viability of cells was determined using the MTT assay, as described above, the mean value of which was considered as 100%. No culture medium was aspirated from Ctrl0 group throughout the entire culture period. The cells in the blank control group (Ctrl) and experimental groups were cultured under the same conditions, except that no Aβ42 was added into the culture or coated onto the plate throughout the culture period in the Ctrl group. The relative adhesion rate of the Ctrl group and experimental groups was calculated by dividing the mean OD490 of adhering cells in each group by the mean OD490 of adhering cells in the Ctrl0 group, and then multiplying by 100%. The relative re-adhesion rate of the Ctrl group and experimental groups was calculated by dividing the mean OD490 of re-adhered (or re-attached) cells in each group by the mean OD490 of adhering cells in the Ctrl0 group and then multiplying by 100%. The relative mortality rate of the Ctrl0 group was ignored, and the relative mortality rate of the Ctrl group and experimental groups was obtained by calculating the difference between 100% and the sum of the relative adhesion and re-adhesion rates of each group.

### 2.4. Analysis of Neurite Outgrowth in the Presence of Extracellular Aβ42 or Its Aggregates

Similarly, the experiments involving extracellular Aβ42 or its aggregates were also divided into two groups (Aβ42-containing DMEM medium or Aβ42-coated plates), as described in the adhesion determination section (Section 2.3), except that the differentiated SH-SY5Y cells were incubated for 24 h at 37 °C with 5% CO_2_ in the presence of extracellular Aβ42 or its aggregates (final concentration: 2.0 μM) with or without HT7 (or HT6) antibody (final concentration: 2.0 μM). Neurites of differentiated SH-SY5Y cells were observed and imaged using an inverted microscopy (IX73, Olympus, Beijing, China). For quantitatively determining the length and number of neurites, the areas of the neurites were extracted using the image analysis software ImageJ (https://imagej.nih.gov/ij/), and the mean length and number of neurites in each group were obtained. Each experiment was repeated in at least three fields (each field contained approximately between 70 and 100 cells) and repeated in five different batches of cells.

In each group, quantitative determination of the length and number of neurites was performed by individuals blinded to experimental conditions. Neurites that emanated from their cell body and had a length equal to or greater than the diameter of their cell body were scored as valid neurites. The average number of valid neurites was obtained by tracing and labeling all valid neurites using the NeuronJ plugin in ImageJ. The average length of valid neurites was obtained by quantifying the length of all valid neurites from their end to the center of their cell body using the Sholl analysis plugin in ImageJ. All data were presented as the mean of at least five batches of data per group. Results were expressed as the mean percentages of the average length and average number of valid neurites, with the mean percentage of the control group as 100%.

### 2.5. Determination of Levels of Palladin in SH-SY5Y Cells in the Presence of Extracellular Aβ42 or Its Aggregates by Dot Blot Assay

The levels of intracellular palladin were analyzed using dot blot and semiquantitative determination by grayscale scanning of the dot images using differentiated and undifferentiated SH-SY5Y cells that were also divided into two groups, as described above in the adhesion/neurite outgrowth analysis section (Section 2.3 and Section 2.4). After culturing in the presence of various extracellular Aβ42 species (final concentration: 2.0 μM) for 24 h, the cells were collected by centrifugation (4 °C, 500 g, 10 min) and were mixed with an appropriate volume of ice-cold lysis buffer [150 mm NaCl, 50 mm Tris-HCl, 1 mm PMSF, 1.0% (*v/v*) Triton X-100, pH 7.4] (C05-01001, Bioss, Beijing, China), followed by ice bath treatment for 5 min and centrifugation for 10 min at 4 °C, 13,680× *g*.

For the dot blot procedure, each lysed sample was directly spotted onto the nitrocellulose membranes through circular templates, and the amount of palladin in each sample was probed by conventional dot blot assay [28]. Palladin and glyceraldehyde-3-phosphate dehydrogenase (GAPHD) were probed with anti-palladin (sc166563, Santa Cruz, Shanghai, China) and anti-GAPDH (bs2188R, Bioss, Beijing, China) antibodies, respectively. All immunoreactive signals were visualized by BeyoECL Moon (P0018FS, Beyotime Biotechnology, Shanghai, China) using secondary HRP conjugated goat anti-mouse IgG (bs0295G-HRP, Bioss, Beijing, China). Finally, the grayscale intensity of the reactive dots was quantitatively determined using image analysis in ImageJ. The relative level of palladin in each dot was obtained by dividing the grayscale intensity of palladin by the grayscale intensity of GAPDH. Finally, the percentage of palladin in each sample was calculated by dividing the mean relative level of palladin in each sample by the mean relative level of palladin in the control and then multiplying by 100%. All experiments were performed in triplicate and repeated five times.

### 2.6. Analysis of Intracellular Palladin Distribution in Differentiated SH-SY5Y Cells and HT22 Cells in the Presence of Extracellular Aβ42 or Its Aggregates by Immunofluorescence Microscopy

The distribution of intracellular palladin in SH-SY5Y/HT22 cells was analyzed by immunofluorescence (IF) microscopy as described previously [29], with slight modification. For palladin IF staining, the cells were incubated with AF488-conjugated anti-palladin antibody (green) (sc166563 AF488, Santa Cruz, Shanghai, China) at 4 °C overnight. We did not validate the specificity of the fluorescence-labeled antibody, based on the manufacturer’s instructions and our previous report, in which the identical anti-palladin antibody without fluorescence label was used [30]. Finally, the IF images were obtained using a laser scanning confocal microscope (Zeiss LSM710, Shanghai, China). Each experiment was performed in at least triplicate and repeated in six different batches of cells.

### 2.7. Statistical Analysis

No a priori sample size calculation was performed. Data were obtained from five or nine different batches of cell and expressed as the mean ± SD (standard deviation). No randomization was used to allocate cells to groups. The cells in each experimental group were measured in random order by two independent investigators. During image capture and analysis, the investigators were blinded to Aβ42 species and the experimental group. A normality test and homogeneity of variance test were performed using the SPSS statistical analysis software. For a dataset, if *p* > 0.05 by the Kolmogorov–Smirnov test, it indicated that the data obeyed a normal distribution, and if *p* > 0.05 by one-way analysis of variance (ANOVA) test, it meant that the data met homogeneity of variance. If the data met normality of distribution and homogeneity of variance, between-group comparisons were performed using Student’s *t*-test analysis, and *p* < 0.05 was considered statistically significant.

## 3. Results

### 3.1. Determination of Adhesion Rates of SH-SY5Y and HT22 Cells in the Presence of Extracellular Aβ42 or Its Aggregates by MTT Assay

**Figure 1:**
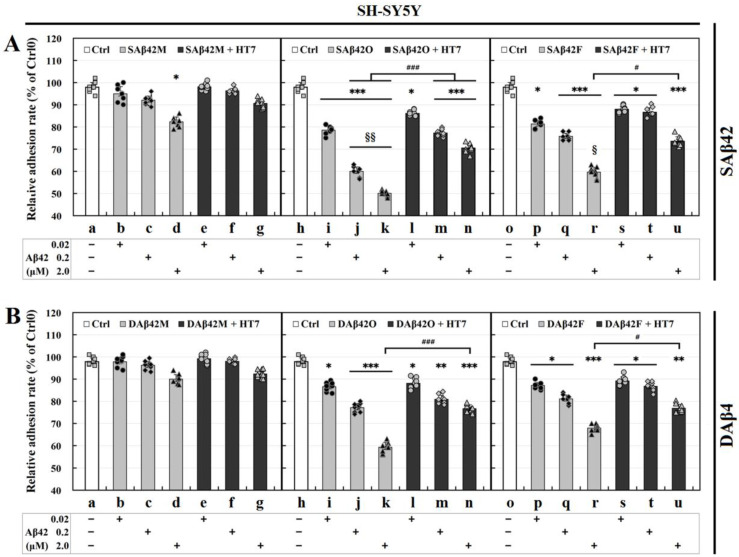

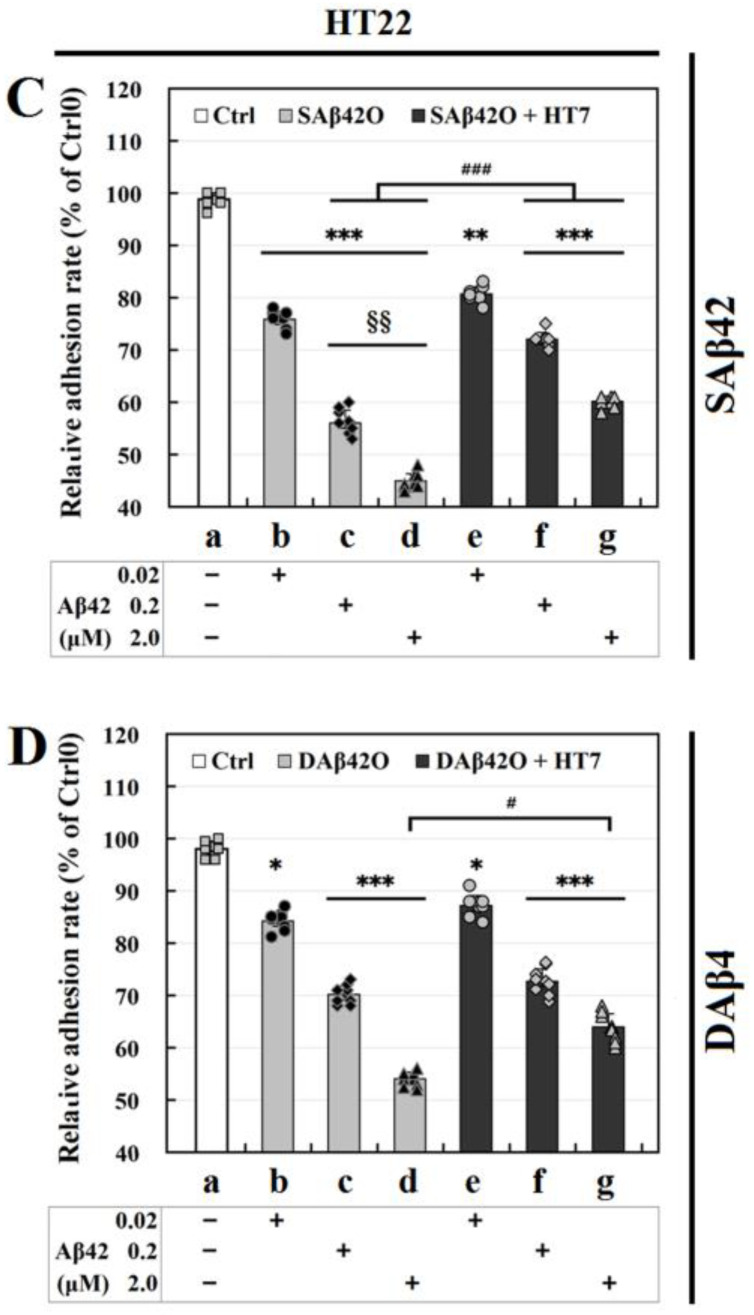
Relative adhesion rates by MTT assay of SH-SY5Y and HT22 cells at 12 h after incubating with three Aβ42 species with/without anti-oligomeric Aβ42 scFv HT7 antibody. (**A**,**B**): SH-SY5Y cells, (**C**,**D**): HT22 cells. Cells in the Ctrl0 (without aspirating shed cells) and Ctrl (aspirating shed cells) groups were cultured normally at 37 °C, 5% CO2 from 12–16 h, and the relative total cell viability in the Ctrl0 group was considered as 100%. Cells in experimental groups were cultured for 12 h in the presence of various SAβ42 and DAβ42 species (final concentration: 0.02–2.0 μM) with or without anti-oligomeric Aβ42 scFv HT7 (or HT6) antibody (final concentration: 2.0 μM). Differences between the Ctrl0 and Ctrl (white columns) or HT7 (or HT6) (not shown) groups were not significant (*p* > 0.05). Asterisks (*) indicate significant differences between experimental and their Ctrl groups (0.01 < * *p* < 0.05, 0.001 < ** *p* < 0.01, *** *p* < 0.001); hash sign (#) indicates significant differences between experimental groups with (black columns) and without (gray columns) HT7 (or HT6) antibody (# *p* < 0.05, ### *p* < 0.001); section break (§§) indicates significant differences between SAβ42 and DAβ42 groups. Each experiment was performed in at least triplicate and repeated nine times with different batches of cells. The data met normality of distribution and homogeneity of variance. All data are shown as mean ± SD.

To determine whether different species and forms of extracellular Aβ42 also have different effects on cell adhesion, and to elucidate the relationship of the anchoring effects of various extracellular Aβ42 aggregates on neural cell adhesion, the adhesion rates of SH-SY5Y and HT22 cells were first measured after they were seeded into 96-well plates at the same density and incubated for 12 h in the presence of the three typical Aβ42 species (monomers, oligomers, and fibrils) in soluble (or suspended) and deposited (or attached) forms. Meanwhile, scFv HT7 (or HT6) antibody was applied to further determine the independent effects of these extracellular Aβ42 aggregates on cell adhesion, since it can inhibit or neutralize the neurotoxicity of Aβ42 aggregates, especially Aβ42 oligomers [26,27]. The adhesion rates of SH-SY5Y and HT22 cell lines in the presence of the three Aβ42 species (monomers, oligomers, and fibrils) within 12 h are shown in Figure 1.

Overall, different species and/or forms of extracellular Aβ42 affect the adhesion of SH-SY5Y cells to varying degrees in a concentration-dependent manner (Figure 1A,B). In the Aβ42M group (*a*–*g* in Figure 1A,B), the cell adhesion rates gradually decreased with the increase in extracellular Aβ42M concentration (from 0.02 to 2.0 μM), but there was no significant difference between the Aβ42M-treated group and the control group (*p* > 0.05), except for the SAβ42M subgroup at high concentrations (2.0 μM; * *p* < 0.05) (*d* in Figure 1A). However, with increasing duration of incubation with Aβ42M but without HT7 (or HT6) antibody, the cell adhesion rates in both the SAβ42M and DAβ42M subgroups, especially the SAβ42M subgroup, were further reduced (SAβ42M/DAβ42M subgroup vs the control group, *p* < 0.05; SAβ42M vs DAβ42M subgroups at 2.0 μM, *p* < 0.05) (data not shown).

Unlike extracellular Aβ42M, extracellular Aβ42O (*h*–*n* in Figure 1A,B) and Aβ42F (*o*–*u* in Figure 1A,B) caused a significant reduction in the cell adhesion rate within 12 h. In the Aβ42O group (*h*–*n* in Figure 1A,B), both SAβ42O and DAβ42O significantly impaired cell adhesion in a concentration-dependent manner (* *p* < 0.05, ** *p* < 0.01, *** *p* < 0.001), and at the same concentration, extracellular SAβ42O caused a greater effect on cell adhesion than extracellular DAβ42O (SAβ42O group vs DAβ42O group, § *p* < 0.05, §§ *p* <0.01) (*i*, *k*, and *n* in Figure 1A,B). This demonstrated that extracellular SAβ42O had a greater impact on cell adhesion than extracellular DAβ42O. This was similar to the case in the Aβ42M group, except that the difference in cell adhesion rates between the SAβ42O and DAβ42O subgroups was more significant. These results indicated that the adhesion of neural cells correlated negatively with the neurotoxicity of extracellular Aβ42O, since SAβ42O showed greater neurotoxicity than DAβ42O [29]. Furthermore, the results from *h*–*n* in Figure 1A,B indicated that the results shown from *a*–*g* of Figure 1A,B should represent the combined effects of extracellular Aβ42M and Aβ42O (freshly formed) on SH-SY5Y cell adhesion, since Aβ42Ms gradually aggregate to Aβ42Os with increasing Aβ42M concentration during incubation at 37 °C. Thus, this also suggested that extracellular Aβ42M, either SAβ42M or DAβ42M, did not actually damage the adhesion of neural cells and might even enhance it.

Similarly, extracellular insoluble Aβ42F dose-dependently caused a significant reduction in cell adhesion (* *p* < 0.05, ** *p* < 0.01, *** *p* < 0.001) (*o*–*u* in Figure 1A,B), and at the same concentration, extracellular SAβ42F showed a greater reduction in the cell adhesion rate than extracellular DAβ42F, especially at high Aβ42F concentration (§ *p* < 0.05), although extracellular Aβ42F showed slightly lower overall negative effects on cell adhesion than extracellular Aβ42O. This was apparently consistent with their correlation with neurotoxicity [29].

Additionally, the results from *e*–*g*, *l*–*n*, and *s*–*u* of Figure 1A,B showed that anti-oligomeric Aβ42 scFv HT7 (or HT6) antibody was able to dose-dependently defend the adhesion capability of SH-SY5Y cells (# *p* < 0.05, ### *p* < 0.001). This meant that by specifically targeting extracellular Aβ42 aggregates, especially Aβ42O, HT7 (or HT6) antibody could dose-dependently block their adverse effects on neural cell adhesion, in addition to protecting cell survival and motility [26,27,29].

In addition to the SH-SY5Y cell line, the HT22 cell line is another in vitro model of neural cells. The HT22 cell line is derived from a primary mouse hippocampal neuronal culture and possesses more neuronal characteristics and phenotypes. To confirm the effect of extracellular SAβ42/DAβ42 or their aggregates on neuronal adhesion, the adhesion rate of HT22 cells was also measured under the same conditions as above. The results showed that overall, extracellular SAβ42/DAβ42 aggregates caused similar but greater decreases in the adhesion rate of HT22 cells than SH-SY5Y cells did. Figure 1C,D shows the adhesion rates of HT22 cells in the Aβ42O group (data of Aβ42M and Aβ42F groups not shown). This indicated that HT22 cells might be more sensitive than SH-SY5Y cells to extracellular Aβ42 aggregates, at least in terms of adhesion.

Overall, the results of the SH-SY5Y/HT22 cells consistently demonstrated that different species and forms of extracellular Aβ42 had different effects on neural cell adhesion, and their effects were directly associated with their neurotrophicity or neurotoxicity; extracellular SAβ42 aggregates adversely affected neural cell adhesion more than extracellular DAβ42 aggregates, especially at higher concentrations. Clearly, the adverse effects of extracellular SAβ42 and DAβ42 aggregates on neural cell adhesion are inversely correlated with their anchoring effects, as reported by Zhang [29].

### 3.2. Extracellular Aβ42 Aggregates Lead to Live De-Adhesion of SH-SY5Y Cells in Addition to Their Death

**Figure 2:**
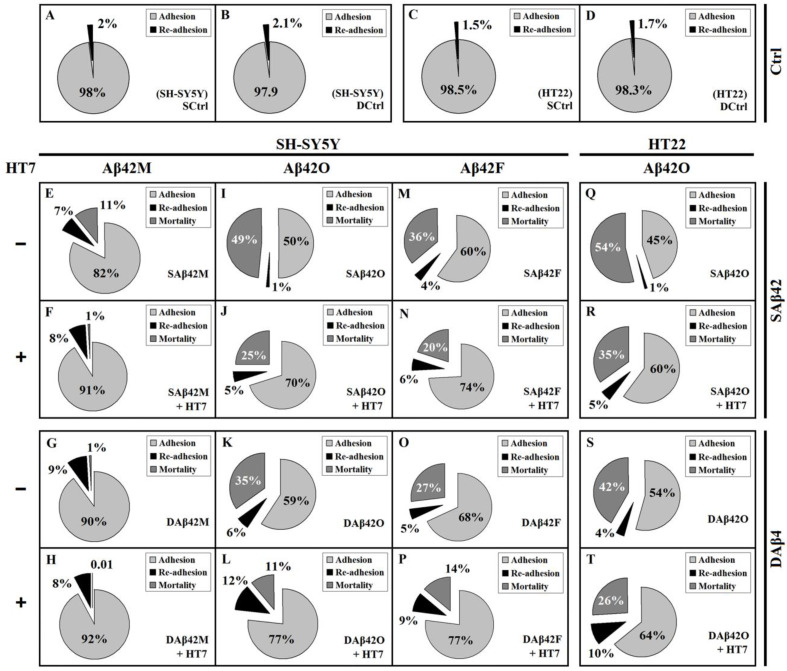
Relative adhesion, re-adhesion, and mortality rates of SH-SY5Y and HT22 cells in the presence of various extracellular SAβ42 and DAβ42 species with/without anti-oligomeric Aβ42 scFv HT7 (or HT6) for 12 h. The relative adhesion rates were from the data of Figure 1. The relative re-adhesion rates were determined by MTT assay after between 4 and 6 h of re-culture of the shed cells in normal 10% DMEM medium at 37 °C and calculated based on 100% of the relative total cell viability in the Ctrl0 group (without aspirating shed cells). The relative mortality rate was calculated by the difference between 100% and the sum of the relative adhesion and re-adhesion rates. The relative mortality rate of the Ctrl group was too small and was ignored. (**A**–**D**): SCtrl and DCtrl of SH-SY5Y and HT22 cells, corresponding to *a* in Figure 1. (**E**–**H**): corresponding to *d* and *g* in Figure 1A,B. (**I**–**L**): corresponding to *k* and *n* in Figure 1A,B. (**M**–**P**): corresponding to *r* and *u* in Figure 1A,B. (**Q**–**T**): corresponding to *d* and *g* in Figure 1C,D.

Given that the adhesion of SH-SY5Y/HT22 cells was positively correlated with the neurotrophicity of extracellular Aβ42M and negatively correlated with the neurotoxicity of Aβ42 aggregates, especially SAβ42O (Figure 1), it was clear that any extracellular Aβ42 (including all species and forms) had consistent effects on the adhesion and viability of neural cells. To determine affiliation of adhesion of neural cells to their viability, the rates of re-adhesion of cells, i.e., the rates of de-adhesion of living cells, in all of the above groups were measured, and the corresponding mortality rates were calculated based on their adhesion and de-adhesion rates. Figure 2 shows the relative adhesion, re-adhesion, and mortality rates of SH-SY5Y/HT22 cells in the presence of 2.0 μM extracellular Aβ42. The results shown in Figure 2E–P demonstrated that there were a certain number of living cells, as well as dead cells, in the shed (or debonded) cells of each group, after exposure to various extracellular Aβ42 for 12 h. Apparently, these living cells (re-adherent cells) belonged to the cells that de-adhered alive, and represented those cells that were only slightly damaged, at least in adhesion, but were able to survive after being transferred to normal culture medium. Inevitably, in addition to the difference in adhesion rates, there were also significant differences in re-adhesion and mortality rates between the various Aβ42-treated groups.

In the Aβ42M group (Figure 2E–H), the vast majority of shed cells were living cells rather than dead cells, except for the SAβ42M subgroup (Figure 2E), where the slightly higher mortality rate was likely caused by a small amount of Aβ42O (freshly formed by Aβ42M aggregation). Apparently due to the beneficial support of Aβ42M and the protection of HT7 (or HT6) antibody, these de-adhered cells were able to stay alive for a period of time, such as 12 h, and re-adhere in order to grow. However, the case was quite different in the Aβ42O group (Figure 2I–L), where the vast majority of shed cells belonged to dead rather than living cells (re-adherent cells), except for the DAβ42O plus HT7 subgroup (Figure 2L), where the number of dead cells and number of re-adherent cells were nearly equal. The re-adhesion and lower mortality rates in the Aβ42F group (Figure 2M–P) were similar to those in the Aβ42O group, which suggested that the neurotoxicity of extracellular Aβ42 aggregates was truly reflected in the actual mortality rate of neural cells, rather than in their total de-adhesion (or debonding) rate.

Similar results were obtained from HT22 cells; however, the mortality rate in the HT22 cell system was higher than that in the SH-SY5Y cell system (*p* < 0.05 to *p* < 0.001). Figure 2Q–T shows the relative adhesion, re-adhesion, and mortality rates of HT22 cells in the presence of extracellular SAβ42O/DAβ42O at 2.0 μM with or without HT7 (or HT6) antibody (data of HT22 cells in the presence of extracellular Aβ42M and Aβ42F not shown). The similar re-adhesion rate and higher mortality rate in the HT22 cell system than in the SH-SY5Y cell system indicated that HT22 cells are more susceptible to the toxic damage of extracellular Aβ42O, which was consistent with the results shown from *h*–*n* of Figure 1A,B and from *a*–*g* of Figure 1C,D.

The results of Figure 2 showed that, overall, both adhesion and re-adhesion rates of neural cells negatively correlated with the neurotoxicity of extracellular Aβ42 aggregates, and the actual mortality rate of neural cells positively correlated with their neurotoxicity. Furthermore, the results of Figure 2 demonstrated again that extracellular SAβ42 aggregates had a greater adverse effect on neural cell adhesion than extracellular DAβ42 aggregates. By comparing the effects of extracellular SAβ42 and DAβ42 aggregates on the adhesion, re-adhesion, and mortality rates of target cells, we found that the greater the neurotoxicity of the extracellular Aβ42 species, the more significant the differences in their damage to neural cell adhesion between their SAβ42 and DAβ42 forms. The presence of re-adherent cells also demonstrated that the damage of neural cells by extracellular toxic Aβ42 aggregates was progressive. Therefore, the higher the toxic activity of the extracellular Aβ42 aggregates, the shorter the survival duration of the cells that were de-adhered alive and the faster they progressed towards death, which inevitably led to a higher mortality rate of the target cells. Additionally, the presence of re-adherent cells also implied that the damage of the neurotoxic Aβ42 aggregates to the cytoskeleton system of neural cells might be more initial or more direct than their damage to the overall cell metabolism. Evidently, the proportion of re-adherent cells shown in Figure 2 represented the proportion of the cells that might be healed or repaired after being damaged (at least in terms of adhesion capacity) by extracellular toxic Aβ42 aggregates. Inevitably, the ultimate fate of these re-adherent cells depended on numerous factors, both inside and outside of the cells.

In addition, it was found that extracellular DAβ42F-HT7 always showed a slightly greater negative effect on the de-adhered cells (lower re-adhesion rate and higher mortality rate) than extracellular DAβ42O-HT7, which might be because the large DAβ42F/DAβ42F-HT7 units physically blocked normal interactions between neural cells and ECM more than the small DAβ42O/DAβ42O-HT7 units, which was directly reflected in their effect on neural cell adhesion(HT7/HT6 alone did not affect the cell adhesion rate, Figure 1). Furthermore, comparisons of the results between *h*–*n* of Figure 1A,B and *a*–*g* of Figure 1C,D and between Figure 2E–H and M–P implied that neuronal cells, especially those in the hippocampus, might be more sensitive to the neurotoxicity of extracellular Aβ42O, which was consistent with our previous report [29].

### 3.3. Effects of Extracellular Aβ42 or Its Aggregates on Neurite Formation and Extension in Differentiated SH-SY5Y Cells

**Figure 3:**
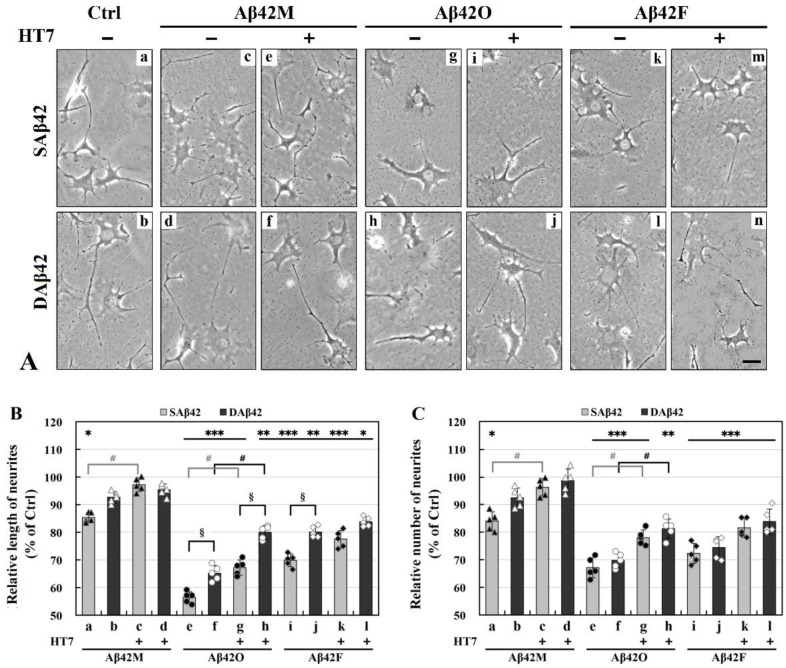
Analysis of neurite outgrowth of differentiated SH-SY5Y cells in the presence of extracellular Aβ42 or its aggregates with or without HT7 for 24 h. (**A**) Representative images of differentiated SH-SY5Y cells. The images represent enlarged partial regions of the representative whole images shown in Figure S1. No difference between control and HT7 alone (not shown). (**B**,**C**) Relative length and number of neurites of differentiated SH-SY5Y cells, with those in the control group as 100%. * *p* < 0.05, ** *p* < 0.01, or *** *p* < 0.001, between Aβ42-treated and the corresponding control groups. § *p* < 0.05, between SAβ42O/F- and DAβ42O/F-treated subgroups. # *p* < 0.05, between Aβ42O/F-treated groups with and without HT7 (or HT6) antibody. Differences between the Ctrl and HT7 (or HT6) (not shown) groups were not significant (*p* > 0.05). Scale bar = 200 μm.

In addition to regulating neural cell adhesion, ECM also regulates neural cell morphology, including neurite outgrowth (neuritogenesis) [31]; meanwhile, neural cell adhesion also directly affects neurite outgrowth. Given that different species and forms of extracellular Aβ42 exerted different effects on the adhesion of SH-SY5Y/HT22 cells, in order to elucidate the correlation between changes in neurite outgrowth and cell adhesion in the presence of extracellular Aβ42 or its aggregates, it was necessary to further investigate the effects of extracellular Aβ42 or its aggregates on neurite outgrowth. This required the use of appropriately differentiated model cells to accurately represent what may be occurring in neurites in vivo. The differentiated SH-SY5Y cells are a useful experimental model for the study of neurite outgrowth because they undergo differentiation to a neuron-like phenotype. The appropriately differentiated SH-SY5Y cells were first prepared and their typical neuronal morphology (with numerous long, branching neurites) was examined by preliminary experiments. Following this, Aβ42-treated differentiated SH-SY5Y cells were imaged, and the length and number of their neurites were quantitatively and statistically analyzed. The representative whole images of differentiated SH-SY5Y cells after 24 h of incubation with various extracellular SAβ42 and DAβ42 species for 24 h are shown in Figure S1, and their enlarged partial images and the quantitative results of the length and number of neurites of the cells are shown in Figure 3.

It can be seen from Figure S1 that, overall, the length and/or number of neurites in each neuron were shortened and/or reduced to varying degrees after 24 h of treatment with various extracellular aggregates. The enlarged partial images shown in Figure 3A are representative of the morphology of the cells in each group. During this period, the control cells formed numerous long neurites and showed normal differentiation patterns (*a*,*b* in Figure 3A), and their shape closely resembled the morphology of neurons in vivo. Compared with the control cells, some, although not all, Aβ42-treated cells (*c*,*d*, *g*,*h*, and *k*–*l* in Figure 3A) exhibited dramatically shortened neurites and/or reduced number of neurites, indicating impaired neurite outgrowths; however, the presence of HT7 significantly mitigated these impairments. Specifically, in the Aβ42M group (*c*–*f* in Figure 3A), the length and number of neurites did not appear to be significantly different from those in the control group (*a*,*b* in Figure 3A), especially in the presence of HT7 antibody (*e*–*f* in Figure 3A). However, in the Aβ42O (*g*–*j* in Figure 3A) and Aβ42F groups (*k*–*n* in Figure 3A), the length and number of neurites were significantly reduced, especially in the absence of HT7 (*g*,*h* and *k*,*l* in Figure 3A). Furthermore, we noticed that the impairment of neurites occurred mainly in their length in the SAβ42 group, and in their number in the DAβ42 group, especially in the Aβ42O group.

Figure 3B,C shows the quantitative data of the length and number of neurites in the corresponding groups shown in Figure 3A, based on all images obtained from five replicate experiments with different batches of cells. As shown from *a*–*d* in Figure 3B,C, after 24 h of cell culture in the presence of extracellular Aβ42M with or without HT7 antibody, there were no significant differences in neurite length and number between most Aβ42M-treated and control (100%) groups, except for in the SAβ42M-treated group (* *p* < 0.05). Similarly, extracellular Aβ42M presumably did not affect neurite occurrence and extension, and the slight reductions in both neurite length and number in the SAβ42M subgroups (*a* in Figure 3B,C), and perhaps in the DAβ42M subgroup (*b* in Figure 3B,C), were presumably caused by a small amount of Aβ42O, newly formed by Aβ42M aggregation during the 24-h incubation period, which could be demonstrated by the significant differences between *a* and *c* in Figure 3B,C (#*p* < 0.05). Anti-oligomeric Aβ42 antibody HT7 (or HT6) has been reported to exert neuroprotective efficacy by specifically targeting Aβ42O [26,27]. Therefore, this also suggested that extracellular Aβ42O might have a direct blocking or inhibitory effect on the neuritogenesis of neural cells, and extracellular SAβ42O was likely to have a greater negative effect on neurite occurrence and extension than extracellular DAβ42O, although there was no significant difference between *a* and *b* in Figure 3B and C during the 24-h period.

However, the quantitative data from *e*–*h* of Figure 3B,C showed that most of the cells treated with either extracellular SAβ42O/DAβ42O and SAβ42F/DAβ42F failed to achieve a normal length and/or number of neurites, indicating that extracellular Aβ42 aggregates caused a dramatic decline in neurite length and number (* *p* < 0.05, ** *p* < 0.01, *** *p* < 0.001); similarly, the damage effects of these extracellular Aβ42 aggregates could be inhibited or blocked to some extent by the anti-oligomeric Aβ42 antibody HT7 (# *p* < 0.05). Evidently, the damage effects of the extracellular Aβ42 aggregates on neurite occurrence and extension were positively correlated with their neurotoxicity. On the other hand, within the same period (e.g., 12 h), the protective efficacy on neurite outgrowth of HT7 (or HT6) antibody at equimolar levels to extracellular Aβ42 appeared to be limited compared to its protective efficacy on cell adhesion (### *p* < 0.001, # *p* < 0.05, *k* and *n, r* and *u* of Figure 1A,B), which suggested that, in addition to the biological effects of extracellular Aβ42 aggregates, their physical barrier effects might also be responsible for blocking or disrupting neurite outgrowth. Further, the results from *e*–*j* of Figure 3B showed that there was a significant difference in neurite length between the SAβ42 and DAβ42 subgroups in these three pairs (§ *p* < 0.05). This suggested that extracellular SAβ42 aggregates significantly affected neurite extension (or growth) in particular. In contrast, there was no significant difference in the number of neurites between these subgroups (Figure 3C). Since the cell adhesion rate in the SAβ42O/SAβ42F subgroup was significantly lower than that in the DAβ42O/DAβ42F subgroup (*k* and *r* in Figure 1A,B, *d* in Figure 1C,D), the average number of neurites per neural cell in the DAβ42O/DAβ42F subgroup should actually be lower than that in the SAβ42O/SAβ42F subgroup.

The images and quantitative data in Figure 3 demonstrate that extracellular Aβ42 aggregates have complex and diverse adverse effects on neurite outgrowth through their biological effects and perhaps their physical barrier effects, in which SAβ42 aggregates appeared to mainly affect neurite extension, while DAβ42 aggregates appeared to mainly affect neurite occurrence. This implied that extracellular SAβ42 and DAβ42 aggregates adversely affect neurite outgrowth through similar but not identical mechanisms, and thus, damaged neurites in different ways. As a result, however, damage to either of these two aspects would eventually damage the neurite network.

### 3.4. Dynamic Changes in Levels and Subcellular Localization of Scaffold Protein Palladin

(1) Determination of intracellular palladin level in differentiated and undifferentiated SH-SY5Y by dot blot assay

**Figure 4:**
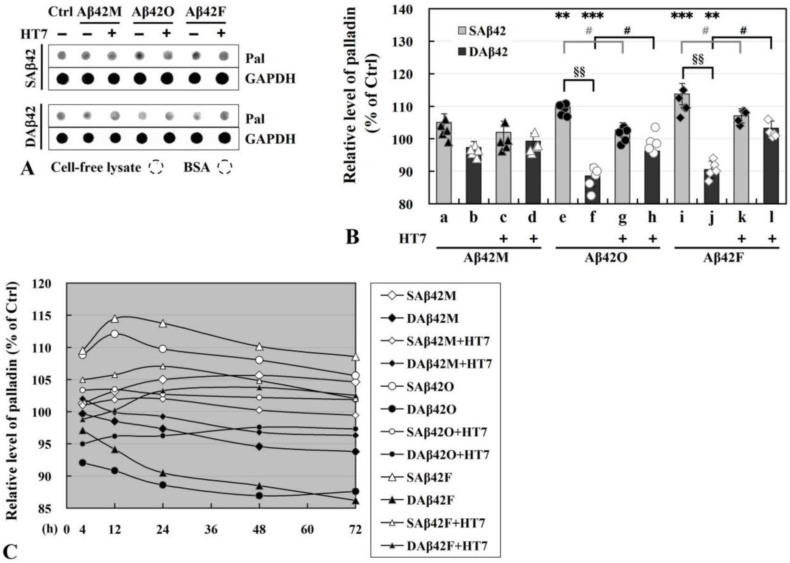
Analysis of palladin level in differentiated SH-SY5Y cells in the presence of extracellular Aβ42 or its aggregates with or without anti-oligomeric Aβ42 antibody HT7 (or HT6) for 24 h. (**A**) Representative dot images for the analysis of palladin levels in the lysate supernatants of differentiated SH-SY5Y cells by dot blot assay after 24 h of culture. Approximately 10 μg of protein was loaded per dot. Pal, palladin; Ctrl, control; BSA, bovine serum albumin. The percentage of palladin level in the control group in (**B**,**C**) was considered as 100%. (**B**) Percentages of palladin levels calculated by quantitative grayscale analysis of the corresponding dots in (**A**). ** *p* < 0.01 or *** *p* < 0.001, between Aβ42-treated and the control groups. §§ *p* < 0.01, between SAβ42O/SAβ42F and DAβ42O/DAβ42F subgroups. # *p* < 0.05, between Aβ42O/Aβ42F groups with and without HT7 (or HT6) antibody. (**C**) Time-courses of the percentages of palladin levels in all groups from 4 to 72 h. The legend on the right indicates the name of each group. The error values of all experimental points were within 5%, not shown on the curves.

**Figure 5:**
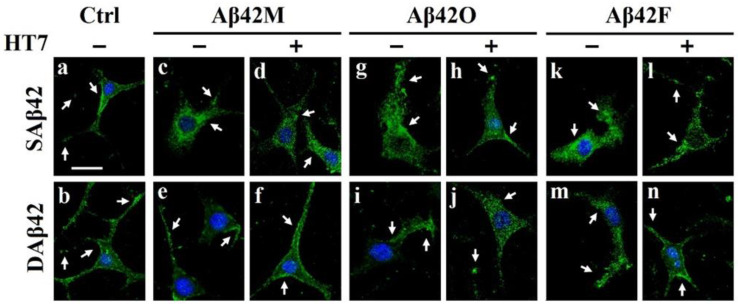
Representative confocal images of immunofluorescence of palladin (green) in differentiated SH-SY5Y cells at 24 h after incubating with three Aβ42 species with/without anti-oligomeric Aβ42 scFv HT7 antibody. These images represent enlarged partial regions of the representative whole images shown in Figure S2. The merged images show palladin (green) and nucleus (blue). White arrows point to significant localization of palladin. Scale bar = 30 μm.

Since intracellular palladin is directly involved in the assembly and construction of the neuronal actin cytoskeleton and directly regulates actin dynamics, it was necessary to determine whether intracellular palladin is affected by extracellular Aβ42 or its aggregates, and if so, how it relates to the detrimental effects of extracellular Aβ42 aggregates on cell adhesion (Figure 1 and Figure 2) and neurite outgrowth (Figure 3). Therefore, we next analyzed the levels of intracellular palladin in differentiated SH-SY5Y cells after 24 h of culture in the presence of extracellular SAβ42 and DAβ42 and their aggregates with or without anti-oligomeric Aβ42 antibody HT7 (or HT6) by dot blot assay (Figure 4) and examined its subcellular localization by IF staining (Figure 5).

As shown in Figure 4A, various species and forms of extracellular Aβ42 caused varying degrees of changes in the levels of intracellular palladin within 24 h. Semiquantitative analysis (Figure 4B) of these dot images by grayscale scanning showed that, surprisingly, extracellular SAβ42 and DAβ42 induced almost opposite changes in the levels of palladin compared to the controls (100%). Clearly, the levels of palladin in the SAβ42O/SAβ42F subgroups were significantly up-regulated (*e* and *i* in Figure 4B), whereas those in the DAβ42O/DAβ42F subgroups were significantly down-regulated (*f* and *j* in Figure 4B) (** *p* < 0.01, *** *p* < 0.001 between Aβ42O/F groups vs control). Consequently, there were significant differences in palladin levels between each pair of DAβ42O/DAβ42F and SAβ42O/SAβ42F groups (§§ *p* < 0.01). These seemingly contradictory results might be the intracellular manifestation of the different effects of extracellular SAβ42 and DAβ42 aggregates on target cells. This indicated that the soluble (or suspended) and deposited (or attached) forms of extracellular Aβ42 aggregates induced different aspects of target cells and facilitated distinct intracellular palladin responses over 24 h. This implied that the mechanisms by which extracellular SAβ42 and DAβ42 aggregates affected neural cells might be very different; after all, the SAβ42 aggregates in ECM exerted their toxic matrix effect through targeting a more spatial neural cell surface, whereas the DAβ42 aggregates in ECM exerted their toxic matrix effect only through targeting a limited neural cell surface. Likewise, similar but slight up-regulation and down-regulation in palladin levels occurred in the SAβ42M and DAβ42M subgroups, respectively (*a* and *b* in Figure 4B), although there was no statistically significant difference between the subgroups or between Aβ42M and the control groups. Similarly, the slight increase or decrease in palladin levels in the SAβ42M or DAβ42M subgroups was presumably induced by a small amount of SAβ42O or DAβ42O (freshly formed by Aβ42M aggregation), respectively.

However, in the presence of HT7 (or HT6) antibodies, both up- and down-regulation of palladin levels in the Aβ42O/Aβ42F-treated groups (*g*, *h*, *k*, and *l* in Figure 4B) were significantly attenuated, and the levels of palladin in these groups were not statistically significantly different from that in the control group. This also demonstrated that extracellular Aβ42O/Aβ42F indeed induced changes (up- or down-regulation) in intracellular palladin levels, but induction could be inhibited or blocked by anti-oligomeric Aβ42 scFv HT7 (or HT6) antibodies.

With reference to previous reports [20], palladin levels are found to rapidly increase in the astrocytes located closest to the wound edge; thus, we speculated that the over-elevated palladin levels in the SAβ42O/SAβ42F groups (*e* and *i* in Figure 4B) might represent a stress response of neural cells to extracellular SAβ42 aggregates, but this stress response did not manifest in the DAβ42O/DAβ42F groups (*f* and *j* in Figure 4B). Although the reason for this difference was not understood here, it at least indicated that the stress-dependent up-regulation of palladin was related to extracellular SAβ42 aggregates. However, this excessive up-regulation of palladin levels in the SAβ42O/F groups apparently failed to truly achieve compensatory improvement of cytoskeletal organization in the target cells; thus, neither neural cell adhesion (*h*–*u* in Figure 1A,C) nor neurite outgrowth (*e* and *i* in Figure 3B,C) were compensatively secured or repaired. This indicated that although palladin plays a critical role in maintaining the integrity of cytoskeleton organization, it is only necessary, not sufficient.

In contrast, down-regulation of palladin levels induced by extracellular DAβ42O/DAβ42F (*f* and *j* in Figure 4B) leads to the decline in the actin cytoskeleton organization, according to previous reports [19,32] that down-regulation of palladin may reduce neuronal adhesion and retard neuronal growth cone formation and neurite outgrowth, which inevitably causes the impairment of cell adhesion and neurite outgrowth. Thus, the down-regulation of palladin levels in the DAβ42O/DAβ42F groups should be largely, if not entirely, responsible for the decrease in cell adhesion (*h*–*u* in Figure 1B,D) and neurite outgrowth (both in length and number) (*f* and *j* in Figure 3B,C). Furthermore, the down-regulation of palladin levels might be one of the manifestations of the toxic effects of extracellular DAβ42O/DAβ42F on neural cells.

To further determine whether these changes in palladin levels in Figure 4B were related to the duration of extracellular Aβ42 action, time-course experiments from 4 to 72 h were performed by dot blot assay for all of the above groups, and the semiquantitative analysis for dot images obtained by grayscale scanning is shown in Figure 4C (dot images not shown). We found that the overall pattern of changes in palladin levels at other time points was almost identical to that at 24 h (Figure 4B), but palladin levels in most groups gradually decreased over time after 24 h. In addition, the palladin levels in the groups with SAβ42F/DAβ42F plus the HT7 antibody (small white/black triangle, Figure 4C) were almost the same after 48 h, which might be related to the easier deposition of large SAβ42F-HT7 particles. Moreover, all of the above analyses of palladin levels were again performed using undifferentiated SH-SY5Y cells, and almost identical results were obtained (data not shown).

Taken together, the results in Figure 4 demonstrated that extracellular Aβ42 aggregates induced changes in the level of intracellular palladin; further, there was a specific or distinct correspondence between the species and form of extracellular Aβ42 aggregates and the resulting intracellular palladin levels, as follows: extracellular SAβ42 aggregates tended to induce an excessive up-regulation of intracellular palladin levels, whereas extracellular DAβ42 aggregates tended to induce a down-regulation of intracellular palladin levels, both of which might affect the integrity of the intracellular cytoskeleton and result in a degenerate and abnormal cytoskeleton system, thereby leading to impairment of neural cell adhesion (Figure 1 and Figure 2) and neurite outgrowth (both in number and length) (Figure 3). Thus, either abnormally elevated or reduced levels of intracellular palladin was detrimental to both neural cell adhesion and neurite outgrowth.

(2) Analysis of subcellular localization of intracellular palladin in differentiated SH-SY5Y cells and HT22 cells by IF staining.

To investigate differences in the subcellular distribution of palladin in the presence of extracellular Saβ42 and Daβ42 and their aggregates, IF staining with fluorescence-labeled anti-palladin antibody and confocal imaging were performed using differentiated SH-SY5Y cells and HT22 cells. After incubating SH-SY5Y cells with various extracellular Aβ42 for 24 h, we observed palladin (green) in the cells, including in the cell body and neurites, by confocal laser scanning microscopy. The representative whole images of SH-SY5Y and HT22 cells are shown in Figure S2A, and their enlarged partial images are shown in Figure 5.

As can been seen from the images in Figure 5, there were some differences in the subcellular distribution of palladin in the different groups. Images of control cells (*a* and *b* in Figure 5) showed that palladin was not uniformly distributed to each region in the cells. Specifically, palladin was concentrated at the growth cone-like or neurite branch sites (middle and right arrows), while it was also intermittently distributed at the periphery of cytoplasmic regions and in puncta along the length of neurites (left arrows; *a* and *b* in Figure 5), consistent with previous reports [19]. This indicated that the distribution of palladin within neural cells had regional characteristics. In the Aβ42M group (*d*, *e*, and *f* in Figure 5), the subcellular localization of palladin was basically the same as that in the control group, except for that in the SAβ42M subgroup, where palladin was mainly distributed in the cytoplasm and only slightly concentrated at the growth cone-like region (*c* in Figure 5). By relating this subcellular localization of palladin with the corresponding results in *d* of Figure 1A and in *a* of Figure 3B,C, it followed that extracellular Aβ42 oligomers (freshly formed) induced an aberrant change in the subcellular localization of palladin in neural cells, but that this induction could be inhibited or blocked by anti-oligomeric Aβ42 antibodies, such as HT7 (*d* and *f* in Figure 5).

However, in the Aβ42O (*g*–*j* in Figure 5) and Aβ42F (*k*–*n* in Figure 5) groups, the difference in the subcellular localization of palladin between the SAβ42O/SAβ42F and DAβ42O/DAβ42F subgroups or between the Aβ42O/Aβ42F subgroups with and without HT7 (or HT6) appeared to be very significant. As shown in the SAβ42O/F subgroups (*g* and *k* of Figure 5), high levels of palladin were present in irregular growth cone-like regions and throughout their disorganized extensions (arrows). Combined with the results in *i*–*k* and *p*–*r* of Figure 1A, in *b*–*d* of Figure 1C and in *e* and *i* of Figure 3B,C, the results of the SAβ42O/SAβ42F subgroups in Figure 5 suggested that the excessive up-regulation of palladin and chaotic distribution of palladin in neural cells might cause a collapse response in neurite outgrowth, which should be one of the reasons for the decreased cell adhesion and neurite outgrowth. However, in the DAβ42O/DAβ42F subgroups (*i* and *m* of Figure 5), much less palladin was observed in slightly irregular growth cone-like regions and their disorganized extension areas, which also corresponded to the declines in cell adhesion (*i*–*k* and *p*–*r* of Figure 1B, *b*–*d* of Figure 1D) and neurites (*f* and *j* of Figure 3B,C). These declines should be caused by defective cytoskeletal organization due to reduced palladin. In contrast, the distributions of palladin in the Aβ42O/Aβ42F plus HT7 subgroups (*h*, *j*, *l*, and *n* in Figure 5) appeared to be modified somewhat; that is, the palladin levels appeared to be more similar to those in the control groups (*a* and *b* in Figure 5).

The results shown in Figure 5 demonstrated that excessive up-regulation of palladin resulted in difficulty in forming efficient growth cones at the appropriate sites, possibly due to cytoskeletal disturbances, resulting in the failure of neurites to grow properly. Conversely, palladin deficiency also resulted in a reduction in available growth cones, resulting in a reduction in the number of efficient long neurites. Additionally, overall, the results in Figure 5 confirmed an increase in the palladin level in the SAβ42O/SAβ42F subgroups and a decrease in the palladin level in the DAβ42O/DAβ42F subgroups, as shown in Figure 4. Apparently, the results of Figure 5 were consistent with those of Figure 1, Figure 2, Figure 3 and Figure 4. These consistencies, taken together with previous reports [19], demonstrated that extracellular Aβ42 aggregates might induce an abnormal change in the level and subcellular distribution of intracellular palladin in neural cells, which could lead to a range of consequences, including the impairment of neural cell adhesion and neurite outgrowth. To examine the relationship of palladin with neurite extension in neuronal cells, the above IF experiments were also performed with the HT22 neuronal cell line. In parallel experiments, almost identical results were obtained in HT22 cells, and the representative IF images of the Aβ42O group are shown in Figure S2B. Taken together, the results in Figure 4 and Figure 5 provided evidence that, at least, optimal intracellular palladin level and subcellular localization were required for the normal adhesion and neuritogenesis of neural cells.

## 4. Discussion

The well-known pathological hallmarks of AD are associated with increased levels of Aβ42. Aβ42 is an amphipathic molecule, so as its level increases, it is easily induced by itself or other molecules to assume misfolded conformation(s) in vivo or in vitro [33,34], leading to self-aggregation to form soluble oligomers and large insoluble fibrils that eventually deposit in the brain as plaques [3]. When Aβ42 aggregates (either soluble or deposited form) seed in the extracellular space in the brain, they are likely to disturb ECM properties and/or components and adversely affect the interactions between neural cells and the ECM, resulting in impaired (or blocked) neurite outgrowth, in addition to reducing cell adhesion. For neural cells, differences in the length and number of neurites are often accompanied by differences in the level and/or distribution of intracellular substances and in the physiological state of the cells [35,36]. Although there is also evidence that scaffold palladin may regulate neurite outgrowth and growth cone motility [19,21], the effects of various extracellular Aβ42 species on the level and distribution of intracellular palladin remain unknown.

Our previous report proposed the anchoring effect of extracellular Aβ42 aggregates on neural cells via integrin receptors [29]. Apparently, these differential reductions in both cell adhesion and motility were consistent with the respective anchoring effects of extracellular DAβ42 and SAβ42 aggregates. Based on this consistency, we proposed that the anchoring effect of extracellular Aβ42 aggregates was also reflected in reducing neural cell adhesion. The anchoring effect and high freeness of SAβ42O/SAβ42F resulted in a relatively greater decrease in neural cell adhesion, while the anchoring effect and high stickiness of DAβ42O/DAβ42F resulted in a relatively smaller decrease in neural cell adhesion (Figure 1 and Figure 2). Therefore, the adhesion due to the anchoring effect of extracellular Aβ42 aggregates should be regarded as ineffective (or inert) adhesion that might be more prevalent in the DAβ42O/DAβ42F groups than in the SAβ42O/SAβ42F groups; this should also be one of the reasons why the migration rates of most DAβ42 groups were lower than those of the SAβ42 groups [29]. Furthermore, Figure 2 clearly showed that the changes in neural cell adhesion and viability induced by extracellular Aβ42 or its aggregates were closely related. These differences in the adverse effects of SAβ42 and DAβ42 aggregates on cell adhesion suggests that different forms (soluble and deposited) of extracellular Aβ42 aggregates might impair neural cell adhesion through similar but not identical mechanisms.

Given the relative higher adhesion rate in the DAβ42 group than in the SAβ42 group, especially at high extracellular Aβ42 concentrations (Figure 1), our findings suggest that neurites tended to extend (or grow) on ECM, favoring cell adhesion. As a result, the number of long, branched neurites was greater in the DAβ42 group than in the SAβ42 group, as shown in Figure S1 and Figure 3. Thus, it could be speculated that effective synapses formed between neighboring cells would be inevitably greater in number in the DAβ42 group than in the SAβ42 group, which might be one of the reasons why SAβ42 aggregates were more neurotoxic than DAβ42 aggregates, although we did not perform statistical calculations for this.

Our findings in Figure 3, Figure 4 and Figure 5 suggest that different species (monomers, oligomers, and fibrils) and forms (soluble/suspended or deposited/attached) of extracellular Aβ42 may affect neural cells through different preferred target(s) on the neural cell surface, thereby resulting in inconsistent intracellular responses, at least in terms of the intracellular palladin response. Based on the previously proposed anchoring role of Aβ42 aggregates in ECM, it could be speculated that soluble (or suspended) Aβ42 aggregates in ECM might have stronger and more diverse biotoxic effects and a weaker physical barrier effect on neural cells, whereas deposited (or attached) Aβ42 aggregates in ECM might have weaker and fewer adverse biotoxic effects and a more prominent physical barrier effect on neural cells, resulting in deposited (or attached) Aβ42 aggregates having a more significant anchoring effect in ECM. In addition, the presence of large complexes such as DAβ42F-HT7 in ECM might still temporarily have a certain physical barrier, even though this antibody is able to neutralize or inhibit toxicity of Aβ42 aggregates to a large extent [27]. This suggested that, in addition to their neurotoxicity, extracellular Aβ42 aggregates, especially large DAβ42 aggregates, had some independent detrimental effects, including physical barrier effect, on neural cell adhesion and neuritogenesis. Since HT7 specifically targets Aβ42 chain’s N-terminal region, it could be speculated that the rigid C-terminal clusters formed between adjacent Aβ42 chains in Aβ42 aggregates was the primary cause of the physical barrier effect.

The scheduling of scaffold palladin is often an initial response within the cell caused by cell–ECM interactions [20]; therefore, altered cell adhesion and neurite outgrowth were presumably the result of altered levels and/or distributions of scaffold palladin. Here, we showed that extracellular Aβ42 aggregates, especially Aβ42O, could disrupt the normal levels and subcellular localization of endogenous palladin (Figure 4 and Figure 5), which should at least be responsible for the reduced or abnormal neurite outgrowth and adhesion in neural cells (Figure 1, Figure 2 and Figure 3). The results of the present study showed that extracellular SAβ42O and DAβ42O induced up-regulation and down-regulation of intracellular palladin, respectively, which seemed paradoxical but led to consistent consequences for cell adhesion and neurite outgrowth. Extracellular SAβ42 aggregates were relatively dynamic, while extracellular DAβ42 aggregates were relatively fixed. We recently reported that extracellular DAβ42 aggregates are more capable of making neural cells inert than extracellular SAβ42 aggregates [29], so the down-regulation of palladin in the DAβ42O/DAβ42F groups (*f* and *j* in Figure 4B) might be one of the consequences of extracellular DAβ42O/DAβ42F causing neural cells to become inert. Since the remodeling of the actin cytoskeleton is thought to contribute to cell adhesion and neurite outgrowth, which largely depends on the regulatory function of palladin, the down-regulation of intracellular palladin in the presence of extracellular DAβ42 aggregates might be one of the mechanisms by which extracellular DAβ42 aggregates impair the dynamics of focal adhesion and neurites through actin dynamics dysfunction [37].

**Figure 6:**
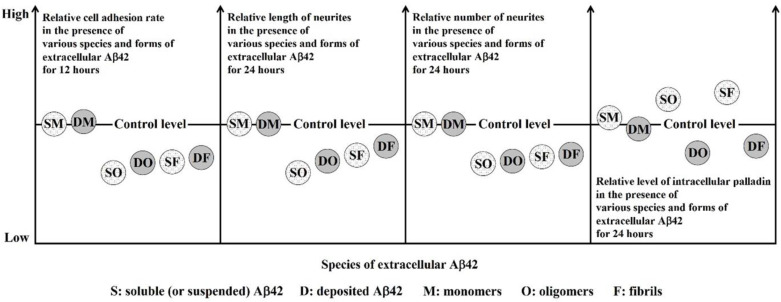
Schematic diagram of the relative effects of various extracellular Aβ42 on neural cell adhesion and in the length and number of neurites and the corresponding relative levels of intracellular palladin. SM, SO, and SF represent soluble (or suspended) Aβ42 monomers, oligomers, and fibrils, respectively. DM, DO, and DF represent deposited (or attached) Aβ42 monomers, oligomers, and fibrils, respectively.

In contrast, the excessive up-regulation of palladin in the SAβ42O/SAβ42F groups might be one of the stress manifestations that occurred as part of the rescue process after target cells were adversely affected by extracellular toxic SAβ42 aggregates; however, our findings suggest that excessive stress up-regulation of palladin was also detrimental to neural cells. A prominent feature of palladin protein is its ability to simultaneously bind multiple regulatory molecules, including enzyme molecules [30,38], so that palladin can modulate multiple cytoskeleton-related cellular events primarily in response to changes in the ECM [39]. Therefore, the excessive up-regulation of palladin might result in feedback to cause multiple cellular stresses, overload the cells with excessive energy consumption, and eventually lead to nerve cell damage or apoptosis due to energy starvation [40]. This might be the reason for the higher mortality rate shown in Figure 2I,Q. On the other hand, it was clear that this stress up-regulation of palladin does not effectively counteract the deleterious effects of extracellular toxic SAβ42 aggregates, at least in terms of cell adhesion or neurite outgrowth. This suggests that regulation by up-regulating palladin in this response was not sufficient, even if necessary, for neural cells, at least in terms of cell adhesion and neurite outgrowth, and perhaps cell migration [29]. Taken together, although palladin was important for cell adhesion and neurite outgrowth, its up-regulation was only beneficial under certain conditions; otherwise, its up-regulation might eventually induce apoptosis, a physiologically programmed process that is tightly spatiotemporally regulated. The scaffold protein palladin seemed to be a “double-edged sword” in regulating cells. Thus, each combination of a certain level and distribution of intracellular palladin produces a unique pattern of neurites (in length and number) in response to a specific species and form of extracellular Aβ42. As for whether the effects of extracellular Aβ42 aggregates were brought on by their toxicity or a general response to oligomeric Aβ42-related stress remains to be further studied. Figure 6 shows the relative effects of various species and forms of extracellular Aβ42 on neural cell adhesion and the length and number of neurite outgrowths and the corresponding relative levels of intracellular palladin.

This study had some limitations. The primary anti-palladin antibody used for immunoblotting and IF staining (Figure 4 and Figure 5) is a monoclonal antibody against the palladin fragment (from 361 to 460 amino acid residues) that can recognize most isoforms of palladin in mammalian cells, but cannot recognize individual isoforms of palladin, including isoform 4 (90 kD), which is an important palladin isoform involved in the regulation of the neural cell cytoskeleton. Thus, these palladin signals in Figure 4 and Figure 5 essentially represented most, but not all, of the palladin in the target cells. To characterize the details of the individual responses of several important palladin isoforms, including isoform 4, to extracellurlar Aβ42 or its aggregates in subsequent studies, antibodies that specifically recognize these palladin isoforms will be required. This is the first task that needs to be carried out for our follow-up study of palladin.

In conclusion, extracellular soluble/suspended or deposited/attached Aβ42 aggregates had differentially detrimental effects on cell adhesion and neurite outgrowth due to the differential responses of intracellular palladin to them. Finally, this study suggests that extracellular Aβ42 aggregates probably act as a variety of extracellular tethering (or anchoring) matrices, and their resulting tethering (or anchoring) effects induce a variety of changes in the intracellular regulatory substances, such as palladin. Certainly, under physiological conditions, the accumulation and deposition of extracellular Aβ42 was presumably very slow, so these tethering (or anchoring) processes were also slow. Therefore, early elimination of the tethering (or anchoring) effects of extracellular Aβ42 aggregates to maintain the regulation and homeostasis of intracellular substances, such as palladin, might be an effective strategy for AD treatment.

## Data Availability

The data presented in this study are available on request from the corresponding author.

## Supplementary Materials

The following supporting information can be downloaded at: https://www.mdpi.com/article/10.3390/biom12121808/s1, Figure S1: Representative images of differentiated SH-SY5Y cells at 24 hours after incubating with three Aβ42 species with/without anti-oligomeric Aβ42 scFv HT7 antibody.; Figure S2: Representative confocal images of immunofluorescence of paladin (green) in differentiated SH-SY5Y cells (A) and HT22 cells (B) at 24 hours after incubating with three Aβ42 species with/without anti-oligomeric Aβ42 scFv HT7 antibody.
