# Supplementary material for: Different Extracellular β-Amyloid (1-42) Aggregates Differentially Impair Neural Cell Adhesion and Neurite Outgrowth through Differential Induction of Scaffold Palladin
Source: Biomolecules. 2022 Dec 2;12(12):1808. doi: 10.3390/biom12121808 (PMC9775237; doi:10.3390/biom12121808)
Supplement: Supplementary file 1 [file biomolecules-12-01808-s001.zip › biomolecules-2010433-supplementary.pdf]

Supplementary figures

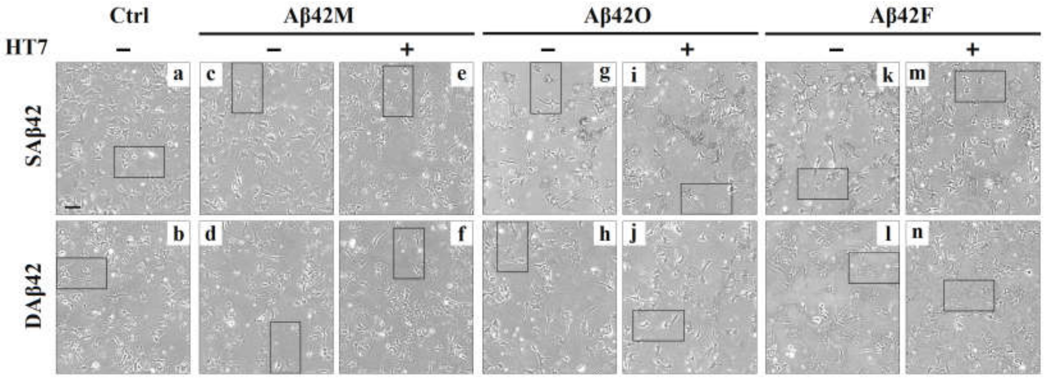

Figure S1. Representative images of differentiated SH-SY5Y cells at 24 hours after incubating with three Aβ42 species with/without anti-oligomeric Aβ42 scFv HT7 antibody.
Ctrl, control; SAβ42, soluble (or suspended) Aβ42; DAβ42, deposited (or attached). Aβ42M, Aβ42O, and Aβ42F represent Aβ42 monomers, oligomers, and fibrils, respectively. Scale bar =100 μm

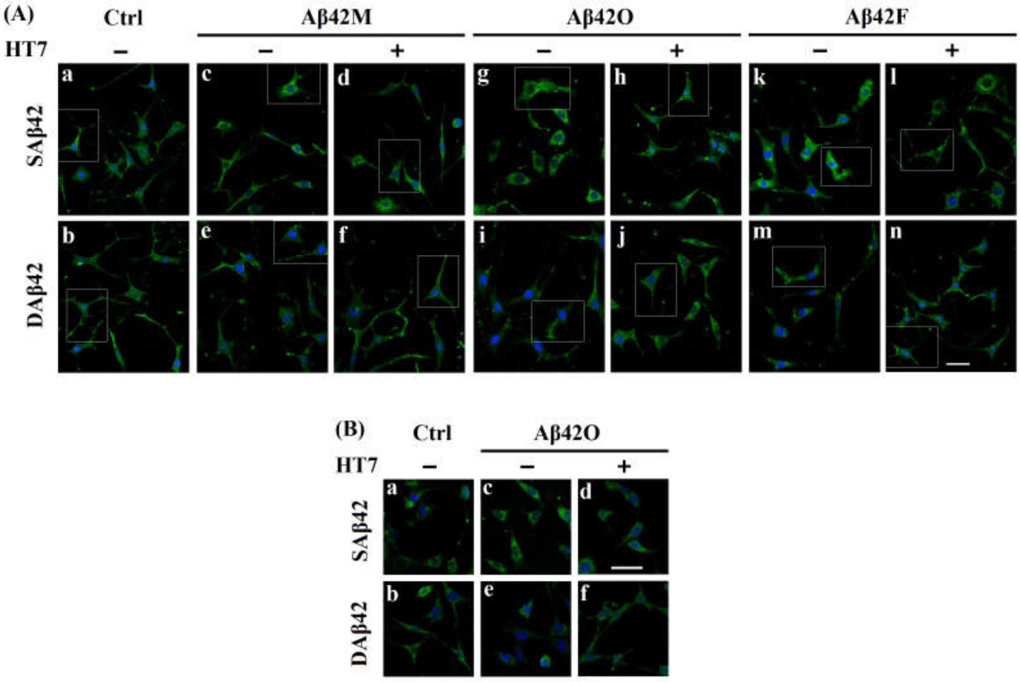

Figure S2 Representative confocal images of immunofluorescence of paladin (green) in differentiated SH-SY5Y cells (A) and HT22 cells (B) at 24 hours after incubating with three Aβ42 species with/without anti-oligomeric Aβ42 scFv HT7 antibody.
After 24 h of culture, the cells were fixed and stained using fluorescent-labeled anti-palladin primary antibody (green). The merged images include palladin (green) and nucleus (blue). Ctrl, control; SAβ42, soluble (or suspended) Aβ42; DAβ42, deposited (or attached). Aβ42M, Aβ42O, and Aβ42F represent Aβ42 monomers, oligomers, and fibrils, respectively. Scale bar =50 μm
